# Optimizing Agroecological Measures for Climate-Resilient Olive Farming in the Mediterranean

**DOI:** 10.3390/plants13060900

**Published:** 2024-03-21

**Authors:** Oumaima Hrameche, Safiye Tul, Ioanna Manolikaki, Nektaria Digalaki, Ioanna Kaltsa, Georgios Psarras, Georgios Koubouris

**Affiliations:** 1 Hellenic Agricultural Organization ELGO-DIMITRA, Institute of Olive Tree, Subtropical Crops and Viticulture, Leoforos Karamanli 167, GR-73100 Chania, Greece; hrameche.oumaima@gmail.com (O.H.); tulsafiye40@gmail.com (S.T.); manolikaki@elgo.gr (I.M.); digalaki@elgo.gr (N.D.); kaltsa@elgo.gr (I.K.); psarras@elgo.gr (G.P.); 2 Mediterranean Agronomic Institute of Chania—MAICh, CIHEAM, Makedonias 01, GR-73100 Chania, Greece

**Keywords:** agroecology, carbon farming, circular economy, climate change mitigation, compost, cover crops, pruning residue, soil moisture

## Abstract

In order to evaluate the potential of climate change mitigation measures on soil physiochemical properties, an experiment based on the application of five agroecological practices such as the addition of composted olive-mill wastes, recycling pruning residue, cover crops, organic insect manure, and reduced soil tillage, solely or combined, was conducted over two years (2020 to 2022) in a 48-year-old olive plantation. The results showed significant increases in soil water content during the spring and summer periods for the combined treatment (compost + pruning residue + cover crops) (ALL) compared to the control (CONT) by 41.6% and 51.3%, respectively. Also, ALL expressed the highest soil organic matter (4.33%) compared to CONT (1.65%) at 0–10 cm soil depth. When comparing soil nutrient contents, ALL (37.86 mg kg^−1^) and cover crops (COVER) (37.21 mg kg^−1^) had significant increases in soil nitrate compared to CONT (22.90 mg kg^−1^), the lowest one. Concerning exchangeable potassium, ALL (169.7 mg kg^−1^) and compost (COMP) (168.7 mg kg^−1^) were higher than CONT (117.93 mg kg^−1^) at the 0–10 cm soil depth and had, respectively an increase of 100.9% and 60.7% in calcium content compared to CONT. Over the experimental period, the implementation of the five agroecological management practices resulted in enhanced soil fertility. In a long-term Mediterranean context, this study suggests that these sustainable practices would significantly benefit farmers by improving agroecosystem services, reducing reliance on synthetic fertilizers, optimizing irrigation water use, and ultimately contributing towards a circular economy.

## 1. Introduction

Climate change is seriously threatening agriculture systems [1], especially in the Mediterranean basin [2]. This major hot spot is one of the most climate-sensitive regions in the world with high vulnerability to the impacts of global warming because it is warming 20% faster than the global average [3,4,5,6]. In fact, the Mediterranean basin is characterized by a semi-arid to arid climate, with hot, dry summers and mild, wet winters [7]. However, this climate is changing rapidly due to global warming caused by human activities, such as the burning of fossil fuels, deforestation, and land-use changes [8]. According to the Intergovernmental Panel on Climate Change (IPCC) [9], the Mediterranean basin is expected to experience significant changes in temperature, precipitation, sea level, and extreme weather events (more frequent and severe droughts and heat waves) in the coming decades. It is expected that the warming will continue with a projected increase of 2.2–4.4 °C by the end of the century depending on the level of greenhouse gas emissions. Also, the annual precipitation is subject to a decrease of up to 30% in some areas. 

These changes are expected to have a great impact on agriculture in general and on crop phenology in particular [10]. They are affecting crop yields, soil health, and water availability, with potential consequences for food security and rural livelihoods [11]. For example, increased soil erosion and degradation, decreased water availability, as well as changes in soil pH and nutrient content are some direct impacts of climate change [12]. Higher temperatures and more frequent and intense droughts are likely to exacerbate these problems, leading to decreased soil fertility and productivity [13]. Furthermore, climate change can also alter the distribution and abundance of pests and diseases, which increase the use of pesticides and other chemicals, and end result in affecting soil quality and overall crop production [14].

Olive trees (*Olea europaea* L.), as an integral part of the Mediterranean landscape and one of its most important socioeconomic tree crops, are also impaired by those changes in the climate [15]. The robustness and plasticity of the olive tree can be illustrated by being a medium–low-income crop, well adapted to hot climate regimes and summer droughts but very sensitive to freezing and snow. Despite its drought tolerance, the extreme aridity is harmful to this crop [16,17]. Regardless of olive trees’ impressive adaptation to the Mediterranean climate [18], global climate change will introduce changes in agricultural ecosystems that will affect photosynthesis and plant productivity [19]. Olive groves represent a huge contribution to providing ecosystem services by protecting soil from erosion and landslides, nutrient cycling, and regulating water, and most importantly the ability to sequestrate carbon [20], particularly in small islands and inland regions where local populations mostly rely on olive-oil production [6]. Consequently, there has been the subject consensus reflecting the potential which olive groves represent in mitigating carbon emissions [20]. 

Overall, the effects of climate change on agriculture, precisely on the cultivation of olive trees in the Mediterranean region, are complex and multifaceted. To effectively mitigate those effects, strategies based on changes in land use and agricultural management aimed at boosting the C stored in the tree biomass and into the soils may be helpful to reduce atmospheric CO_2_ [21,22]. Utilizing crop- and soil-management techniques, that increase soil water storage, minimize the risk of flooding, and alleviate drought-induced agricultural water stress, attenuates the effects of climate change [23]. Locally available organic materials, when recycled, have the potential to enhance carbon storage within soil matrices and furnish mineral nutrients essential for tree growth [24]. Regarding climate change and increasing agricultural needs, soil productivity must be improved or at least maintained [25,26]. Achieving sustained olive productivity in extreme climate conditions necessitates the implementation of Good Agricultural Practices including an improved management of pruning, irrigation, and fertilization techniques, coupled with sustainable soil-improvement methods to mitigate water and nutrient losses in the long term [27]. In fact, the imperative to transition towards sustainable agricultural systems resonates across all levels, from local to global scales. This necessitates the implementation of strategies aimed at enhancing carbon sequestration, mitigating pollutant emissions, and curbing natural-resource depletion, while concurrently ensuring the continued production, quality, and safety of agricultural food [28].

In this context, several agroecological practices can be adopted. As an old practice, cover cropping has a long history of research documenting benefits for farms and the environment. The increased awareness of climate change in recent decades contributes to the resurgence in cover-crop adoption for their ability to reduce erosion [29], fix atmospheric nitrogen, reduce nitrogen leaching, and improve soil health [30]. There is also a body of evidence that supports the ability of cover crops to increase soil carbon or soil organic matter (SOM) [31,32] and to improve the soil’s physical properties which enhance soil–water dynamics [33]. Thus, growing cover crops could reduce soil evaporation and mitigate the adverse impacts of climate extremes during summer, the main crop-growing season [34]. 

Another agroecological practice that is gaining huge attention worldwide, even if it is a traditional practice used for centuries by farmers [35], is the sustainable and climate-smart approach of composting [36]. It has the role of removing waste and transforming it into nutrient-rich organic products at the same time, making it a strong tool for ecological, social, and economic sustainability [37]. These organic products can enhance soil fertility [38], such as crop residues, agroindustry by-products, straw, livestock waste, kitchen waste, and sewage sludge [39]. The addition of composted materials to soils increases the humic substance, a recalcitrant fraction of SOM, which impacts positively the longevity of endogenous SOM [40]. Furthermore, the application of composted organic wastes has a significant increase in the N, P, and K availability and promotes the uptake or stocking of soil residual nutrients in a way to prevent excess nutrients from leaching into the groundwater [41].

Recent studies highlight insect manure as an emerging sustainable alternative to traditional fertilizers due to its nutrient-rich composition and potential benefits for soil health [42]. Also known as frass, it denotes the excreta produced by insects like beetles, caterpillars, and termites, among others [43]. Rich in digested plant matter, microorganisms, and insect waste, it offers essential elements such as nitrogen, phosphorus, and potassium, alongside beneficial microorganisms [44,45]. Its properties are contingent on the insect species and their diet, with varying benefits including enhanced soil structure and microbial activity, promoted plant growth, and improved nutrient availability [44,46]. Renowned for its environmental sustainability due to its minimal carbon footprint and its waste-reduction potential, insect manure finds applications in agriculture as soil amendments, plant fertilizers, and bioremediation agents [47].

With the historical and economic importance of fruit-tree orchards in Mediterranean agriculture, they can be used to mitigate global warming by sequestrating carbon (C) and providing renewable fuels [48]. In most cases, the practice of recycling agricultural waste, which involves techniques like using composted by-products and chopped pruning residue for soil mulching without disturbing the soil mechanically, holds significant promise for improving both fertility and soil biodiversity [49]. Instead of burning and having direct emissions of CO₂ going into the atmosphere, it is much better to proceed to the mulching of pruning residues, thus the soil organic carbon (SOC) and soil fertility are improved. Following appropriate treatment, such as composting, the pruning residues can be incorporated into the soil. This practice yields favorable outcomes for the soil, as it significantly enhances soil carbon levels and overall soil quality [50] and also can be a factor that makes the farmer reduce or omit fertilizer use and the environmental problems associated with residue management.

This study was carried out to evaluate the potential of climate change-mitigation effects through the application of several sustainable soil practices with low negative environmental impact in field conditions on soil properties in a productive olive-grove context. These agroecological practices such as the addition of composted olive-mill wastes, recycling pruning residue, cover crops, organic insect manure, and soil tillage are supposed to influence the soil’s physical and chemical properties.

## 2. Results and Discussion

### 2.1. Effect on Soil Water Content

**Figure 1:**
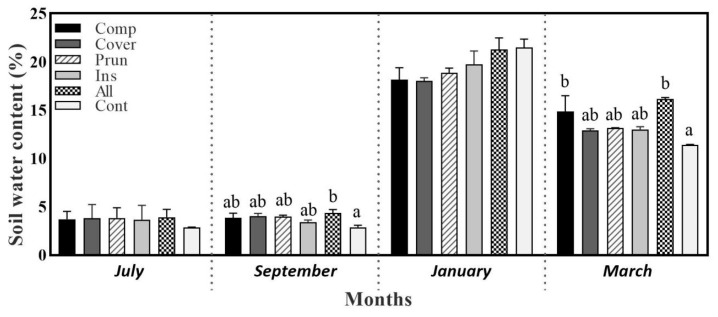
Soil water content at four sampling periods (July—2021, September—2021, January—2022, and March—2022). Mean values ± SE per each treatment are presented. The significance at *p* ˂ 0.05 is indicated using different letters (LSD test).

The soil water content (SWC) was significantly affected by the agroecological practices during March and September, while no significant differences among the treatments were observed in July and January. During September 2021, ALL (4.36%) expressed the highest SWC compared to CONT (2.88%), the lowest, marking an increase of 51.3% in the SWC. The sampling results of March 2022 recorded the highest value in All (16.13%) and COMP (14.86%) compared to CONT (11.39%), marking an increase of 41.6% and 30.4%, respectively (Figure 1). Similar results were found by Devarajan et al. [51], explaining that the introduction of cover crops and composts as soil amendments increased soil moisture. Actually, adding organic matter as compost and manure in olive groves is one of the factors that ameliorate soil permeability and water retention [52,53]. In fact, using compost in the second year increased soil water-holding capacity, which increased SWC, especially in warm periods. For this reason, ALL had the best SWC due to its high content of SOM from compost, pruning residues, and the cover crop for the second year. SWC is a significant indicator of the moisture conditions of soil, reflecting the capacity of soil to hold and supply water, and affecting the movement of nutrients in it, as pointed out by Zhao et al. [54]. In general, SOM, which is made up of approximately 58% soil organic carbon, is associated with an increased water-holding capacity (strong positive correlation) in the surface horizon of agricultural soil [55]. The correlation between SOC and SWC is evident within soil aggregates, as demonstrated by Panagea et al. and Zhao et al. [56,57]. These aggregates are formed through the accumulation of SOM molecules and mineral particles, as elucidated in the study by Abiven et al. [58]. The formation of soil aggregates plays a pivotal role in determining various characteristics such as pore space size, shape, number, and connectivity, as indicated by Strudley et al. and Undawatta et al. [59,60]. The intricate structure of SOM molecules offers numerous sites for ionic binding with soil water, thereby significantly influencing porosity, as highlighted by Schulten and Schnitzer [61]. Additionally, SOM molecules possess a high surface area that is charged, so attracts water, and this one adheres to the surface like static cling. It can retain up to ten times its weight in water [62,63]. 

In more detail, SOM improves the aggregate stability, decreases soil bulk density, increases soil pore volume, and increases the soil’s capacity to hold available plant water [64]. The introduction of SOC facilitated root growth, resulting in decomposition that enhances soil porosity. Coarse roots play a crucial role in enhancing the inter-aggregate pore space [65,66], which significantly increases soil porosity. Consequently, the enhanced soil porosity further contributes to the improvement of the soil water-holding capacity and water-storage capacity. As reported by Bhadha et al. [63], the USDA-NRCS indicates that even the most conservative estimations propose that each 1% rise in SOM can enhance the water-holding capacity of soils by up to 18.71 liters per square meter. The application of agroecological practices can positively affect rainwater infiltration and reduce soil evaporation [67]. As a consequence, less irrigation water is needed to irrigate the olive trees, especially in March and September when water availability for the tree is more important [68].

### 2.2. Effect of the Agroecological Practices on pH and EC

**Figure 2:**
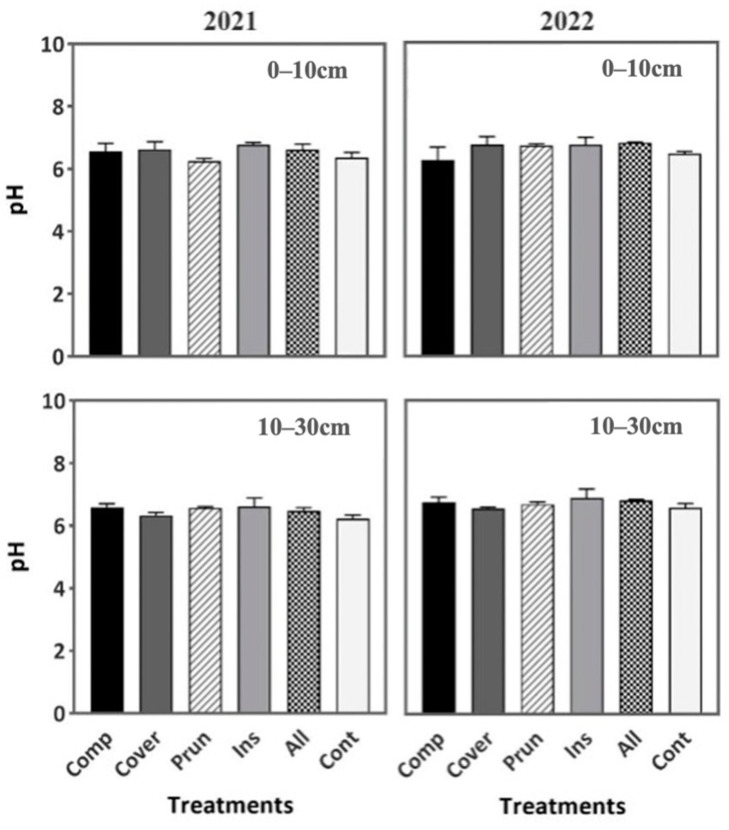
Soil pH during two periods (2021, 2022) at two soil depths (0–10 cm, 10–30 cm). Mean values ± SE per each treatment are presented.

**Figure 3:**
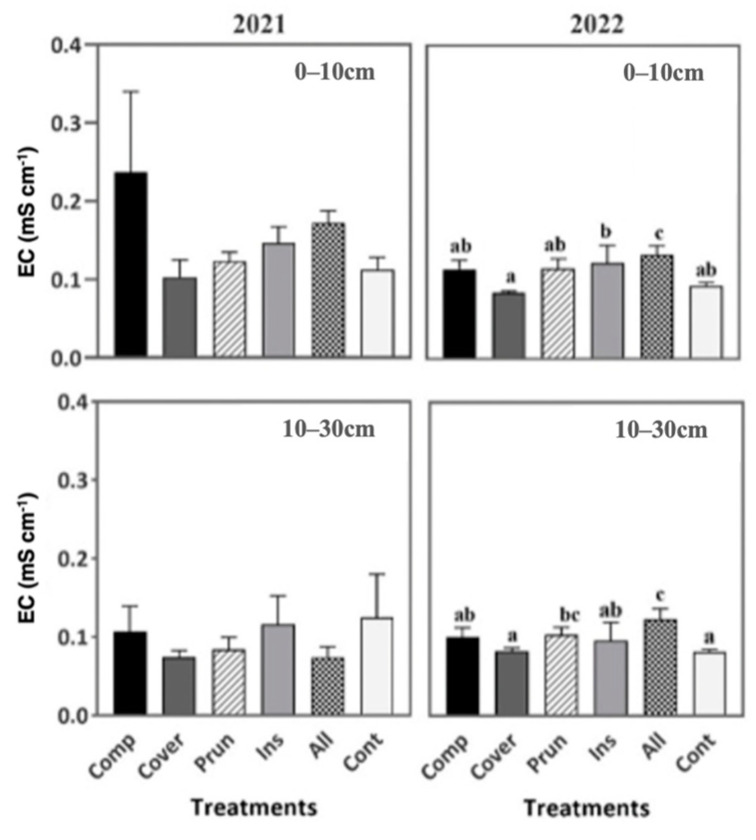
Soil EC during two periods (2021, 2022) at two soil depths (0–10 cm, 10–30 cm). Mean values ± SE per each treatment are presented. The significance at *p* ˂ 0.05 is indicated using different letters (LSD test).

The soil pH, in both years 2021 and 2022, was not significantly affected by the six treatments (Figure 2), while EC showed significant differences among the treatments during the second year in the two soil depths of 0–10 cm and 10–30 cm. ALL recorded the highest EC value (0.13 mS cm^−1^) at 0–10 cm and (0.12 mS cm^−1^) at 10–30 cm compared to the other treatments (Figure 3). A good EC is generally ranked between 0.12 and 0.16 mS cm^−1^, while CONT had a very low EC of 0.09 mS cm^−1^ at 0–10 cm and 0.08 mS cm^−1^ at 10–30 cm (Figure 3). The pH values showed no discernible variation among the treatments, aligning closely with the findings reported by Chehab et al. [69]. The pH value similarities imply that none of the soil agroecological practices applied caused any acidification during the study period [49]. EC is a soil characteristic reflecting indirectly the total concentration of soluble salts and providing directly the salinity measurement [70]. It is worth noting that only in recent times has EC been recognized as a significant variable influenced by agricultural practices and incorporated as a parameter in soil quality indices [71]. The EC of soil exerts a substantial influence on crop suitability, agriculture productivity, the accessibility of essential plant nutrients, and soil microorganisms’ activity [72]. This parameter is influenced by various factors such as irrigation, land use, and the application of fertilizer and compost. Our findings indicate that the EC of soil is undoubtedly influenced by the management practices employed by farmers, regardless of the seasonal conditions and crop phase, as is mentioned by Sharma [73]. Based on Angelova et al. [70], the application of vermicompost and compost to the soil resulted in an upward trend observed in the EC values. This effect can be explained by the direct solubilization of ions and the mineralization of compost that releases soluble mineral nutrients [74].

Since EC has a direct relation with the amount of moisture content of soil particles, it is higher in wet soil compared to dry soil [75]. It makes sense that ALL had the highest EC; it has also the highest SWC, which positively affects the EC.

### 2.3. Effect of the Agroecological Practices on SOM

SOM is a crucial indicator of soil health that is closely linked to agricultural management [76]. It has a beneficial impact on soil physical properties, facilitating water infiltration, preserving soil structure, and enhancing nutrient availability [73].

**Figure 4:**
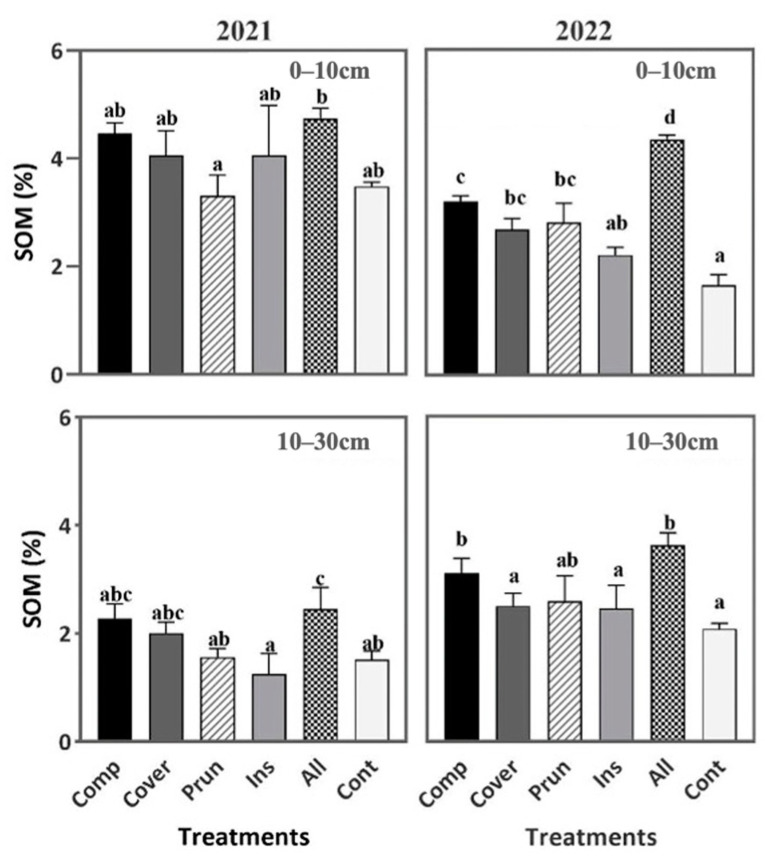
Soil organic matter content during two periods (2021, 2022) at two soil depths (0–10 cm, 10–30 cm). Mean values ± SE per each treatment are presented. The significance at *p* ˂ 0.05 is indicated using different letters (LSD test).

So, when it comes to this parameter, the six treatments were significantly affected. During the first year (2021), ALL expressed the highest value of SOM (4.73%) compared to the lowest values of PRUN (3.30%) and CONT (3.48%) at 0–10 cm, marking an increase of (43.3%) and (35.9%), respectively. At the 10–30 cm soil depth, again ALL showed a significantly higher SOM (2.46%) compared to the lowest values of INS (1.25%), PRUN (1.51%), and CONT (1.56%). During the following year (2022), ALL (4.33%) kept showing statistically higher SOM among the other treatments, followed by COMP (3.20%), marking an increase of 162% and 94% compared to the lowest CONT (1.65%) at 0–10 cm and an increase of (93%) and (49%) compared to the same treatment at 10–30 cm (Figure 4). According to the SOM rankings suggested by Landon (1991), the soil of the experimental field is considered very low to medium (1.25–4.73%) in organic matter content. This significant difference was explained by the combination of chopped tree pruning and compost application [67]. Also, as in the case of ALL, since it contained cover crop, a higher SOM can be achieved by the decomposition of roots, and higher soil cover can lead to higher SOM [77], therefore enhancing soil quality. In another study, it was found that the introduction of cover crops and compost together as soil amendments increased soil macronutrients, organic matter, and soil moisture [51]. In contrast to our findings during the second year, Kavvadias et al. [78] observed that the addition of organic matter solely from compost, shredded pruning residues, or olive mill by-products did not yield a noteworthy impact on SOM. This could be explained by the higher quantity we applied and the continuous application of compost for the second consecutive year resulting in a substantial increase in SOM. Specialized microbial populations use their enzymatic activities to convert organic matter, generating heat that induces physicochemical changes resulting in the production of biomass, CO_2_, and humus-like end-products ([79]. Ultimately, this process yields a stable, complex mixture that is rich in humus, as described by Chowdhury et al. [80].

Based on Figure 4, SOM in the first year was higher at 0–10 cm than the one in the second year at the same soil depth; however, it is essential to mention that when comparing the deeper soil depth of 10–30 cm, the second year had a higher amount of SOM than the first year. This difference in the vertical distribution of SOM between both years is due to the precipitation. After the application of the different treatments during the second year, very strong rainfall occurred during the following month, which led to an infiltration of the SOM and nutrients to deeper soil layers, thus the layer 10–30 cm had a higher percentage of SOM than the year before, marked by poor precipitation.

### 2.4. Effect of the Agroecological Practices on Soil Nutrients

**Figure 5:**
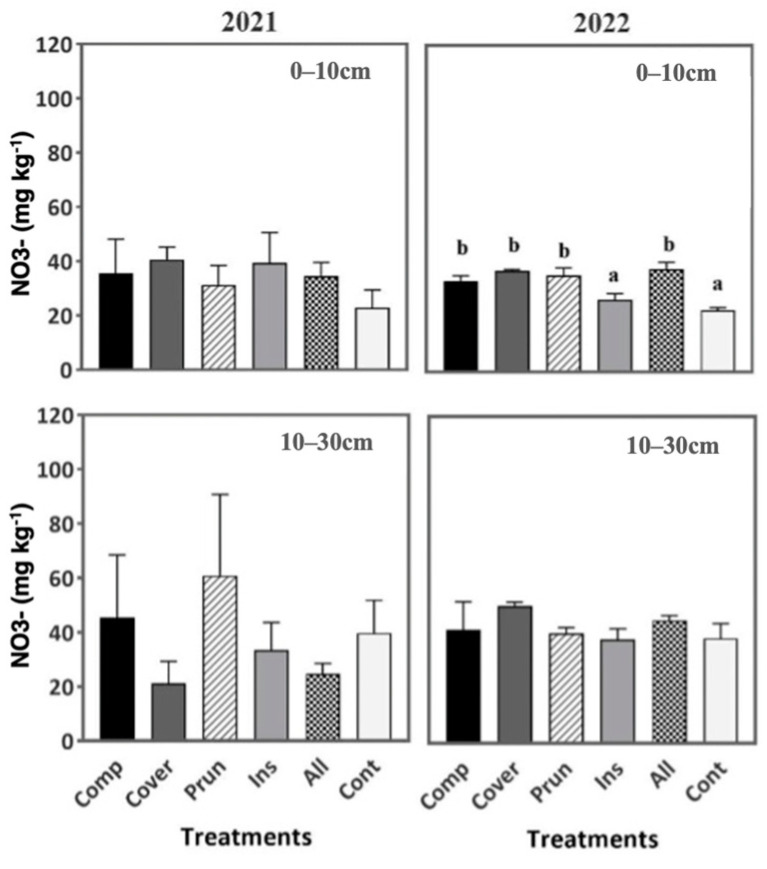
Soil nitrate during two periods (2021, 2022) at two soil depths (0–10 cm, 10–30 cm). Mean values ± SE per each treatment are presented. The significance at *p* ˂ 0.05 is indicated using different letters (LSD test).

None of the treatments had affected nitrate content in the soil during the first year at the two soil depths. It was until the second year (2022) that the differences in soil nitrate were significantly recorded at 0–10 cm; ALL (37.86 mg kg^−1^) and COVER (37.21 mg kg^−1^) had the highest value of NO_3_^−^ and CONT, the lowest one (22.90 mg kg^−1^) (Figure 5). In fact, the greatest output of N by the legumes occurs mainly through the decomposition of its residues [81,82]. Also, Ordóñez-Fernández et al. [83] claimed from their field experiments that legumes can accumulate 85 kg ha^−1^ of NO_3_^−^ in the first 20 cm in the tilled soil of mature olive groves. Zupanc and Justin [84] suggested that plants have access to a significant quantity of N due to the presence of organic matter, an acidic pH, and appropriate soil moisture levels, which is in accordance with our results. 

Also, the amount of NO_3_^−^ did not increase during the second year. This is probably due to the immobilization of N, since the sampling was performed months after the treatment application. Based on Magdich et al. [85], the addition of organic amendments resulted in an increase in total nitrogen in the soil, which was primarily in the organic form. The soil microbial activity transformed the nitrogen content present in olive-mill waste (OMW) and compost, leading to the production of NH_4_^+^, NH_3_, and ultimately NO_3_^−^. This transformation process, along with the improvement in nitrogen content, led to a temporary mineral nitrogen immobilization into the organic form due to telluric microflora. This phenomenon, commonly referred to as “Turnover”, can be advantageous as it represents a stable organic form of nitrogen storage [86,87].

**Figure 6:**
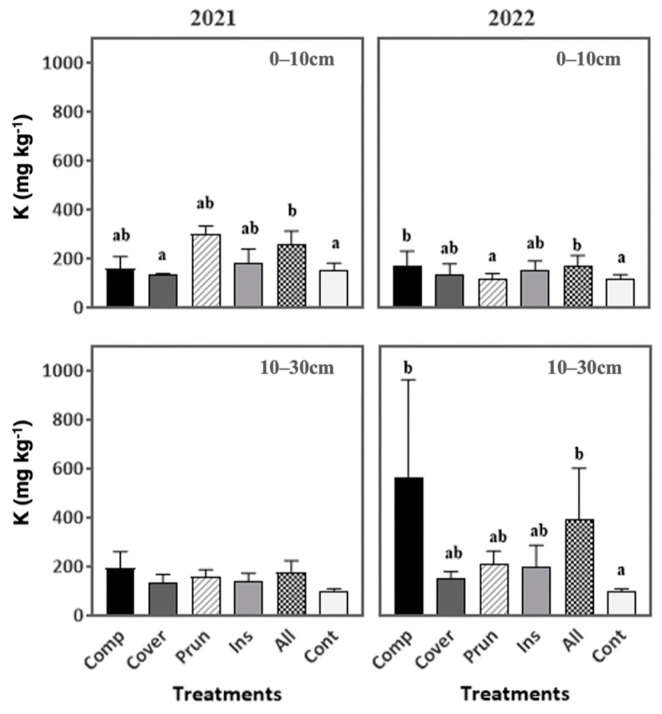
Soil exchangeable potassium during two periods (2021, 2022) at two soil depths (0–10 cm, 10–30 cm). Mean values ± SE per each treatment are presented. The significance at *p* ˂ 0.05 is indicated using different letters (LSD test).

In contrast, the treatments affected the potassium in the soil during both years except at the soil depth of 10–30 cm during the first year, where no significant differences were found. In 2022, the potassium in the soil for ALL (169.7 mg kg^−1^) and COMP (168.7 mg kg^−1^) was higher than CONT (117.93 mg kg^−1^) by 44% and 43%, respectively, at 0–10 cm. Also, at 10–30 cm, ALL and COMP had significantly higher values of potassium than CONT, marking, respectively an increase of 298% and 478% at 10–30 cm (Figure 6). Similar results were found by Angelova et al. [70], where K increased in the soil by the application of vermicompost, and this may be attributed to the vermicompost and compost which have high K contents. Actually, compost addition improved the nutrient cycling in soil organic decomposition processes (enzymatic activities of glucosidase and phosphatase) and soil nutrient availability (total N and extractable K contents) [88]. Also, the increase in K levels in the soil amended with compost and OMW is explained by the richness of compost and OMW in these elements, and also by the organic matter decomposition [89,90,91]. An increase in SOM led to a decrease in K fixation, which in turn increased K availability [92]. Researchers such as Singh al. [93] and Verma et al. [94] have reported that the long-term use of mineral fertilizers, manure, compost, and other soil improvers increases the potassium content in the soil. This is due to the high amount of K found in organic amendments that increase the soil’s CEC, resulting in higher K levels in the soil [70]. The high K levels at 10–30 cm during the second year could be explained by the high concentration of K in OMW and also by the fact that K is a highly soluble element that can easily infiltrate through various soil horizons [95], which is supported in our study by the heavy rainfall after the application of the treatment.

Generally, combining the agroecological practices as in ALL contributes to an increase in some important micro and macronutrients, and more particularly, that of the exchangeable K content, often, a limiting soil nutrient for olive trees [96]. The application of organic amendments to the soil has been found to improve soil fertility and reduce the need for chemical fertilizers. As a result, the soil’s potassium levels increase, which provides valuable drought tolerance, especially in the frequently dry Mediterranean region, as noted by Magdich et al. [85].

**Figure 7:**
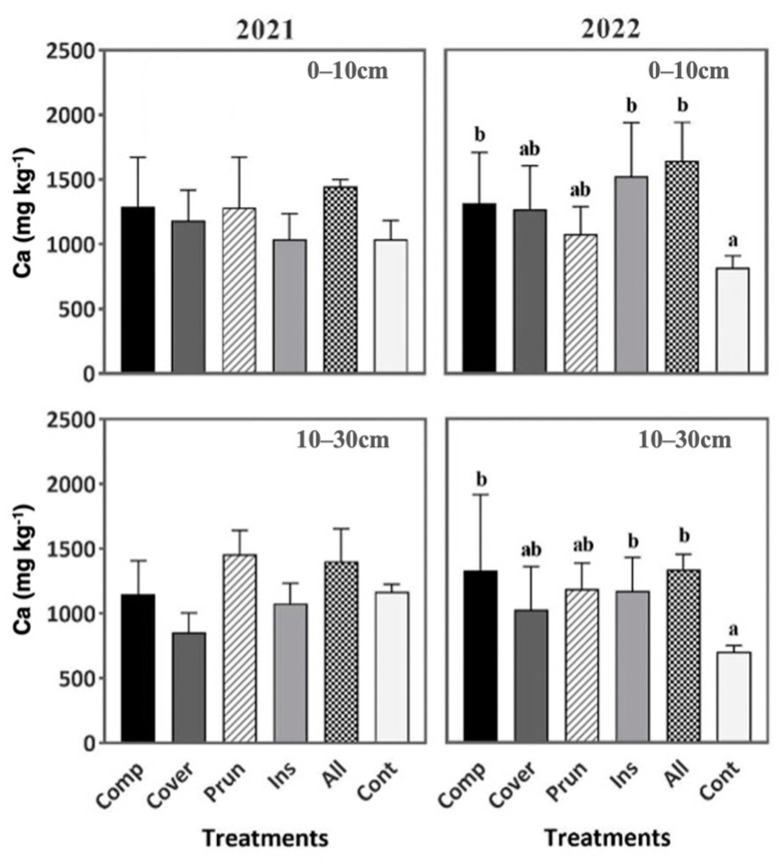
Soil exchangeable calcium during two periods (2021, 2022) at two soil depths (0–10 cm, 10–30 cm). Mean values ± SE per each treatment are presented. The significance at *p* ˂ 0.05 is indicated using different letters (LSD test).

Similar results were found in the calcium content where ALL, COMP, and INS are significantly higher than CONT by 100.9%, 60.7%, and 86% at 0–10 cm and by 90.6%, 89%, and 67% at 10–30 cm, respectively, during the second year. No significant differences were recorded during the first year (Figure 7). Combining different agroecological practices as one treatment as in the case of ALL is more efficient in having an increase in calcium content compared to one practice or none.

**Figure 8:**
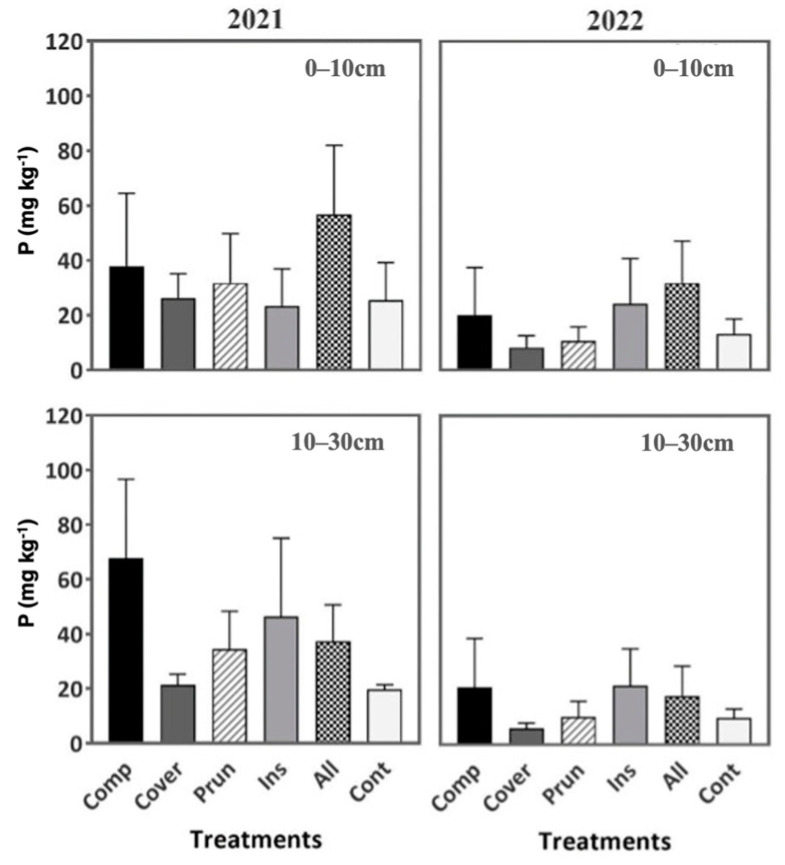
Soil phosphorus during two periods (2021, 2022) at two soil depths (0–10 cm, 10–30 cm). Mean values ± SE per each treatment are presented.

**Figure 9:**
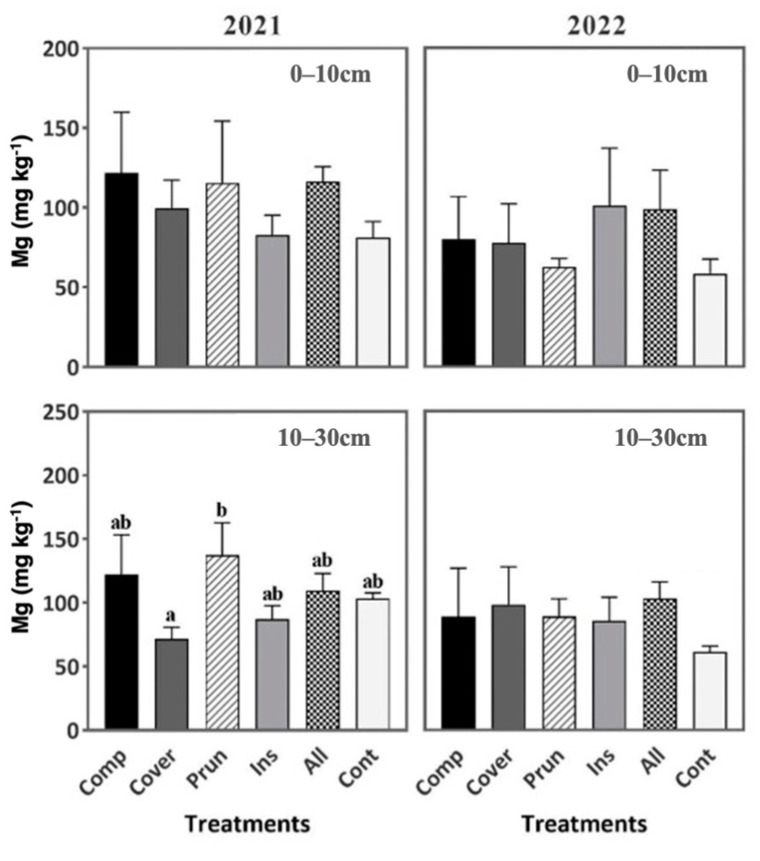
Soil exchangeable magnesium during two periods (2021, 2022) at two soil depths (0–10 cm, 10–30 cm). Mean values ± SE per each treatment are presented. The significance at *p* ˂ 0.05 is indicated using different letters (LSD test).

When comparing the soil content in Magnesium and Phosphorus, it was not affected significantly by the treatments either in the first year or the second year of the study (Figure 8 and Figure 9). This result can be attributed to the OMW low phosphorus concentration (0.74 g L^−1^) [85]. According to Nasini et al. [41], an increase in phosphorus within soil layers 0–15 and 15–30 cm was observed in olive groves after the forth year of spreading solid waste from olive-oil extraction.

The six treatments showed an available P level classified as medium to high; 5–15 mg kg^−1^ as medium and >15 mg kg^−1^ as high based on Landon [97]. Generally, the soil P of ALL and COMP are the highest during both years and the two soil depths. Thus, good organic matter mineralization is a result of the different organic matter sources supplied as inputs and the high soil humidity. These high values compared to CONT might be due to dynamic nutrition flows as a result of organic matter inputs. Indeed, the incorporation of compost can elevate soil nutrient levels by introducing carbon and fostering organic matter accumulation, as indicated by Wichuk et al. [98] and Wei et al. [99]. This practice contributes to the establishment of an extensive reservoir of stored nutrients, thus promoting long-term soil fertility maintenance, as emphasized by Liu et al. [100] and Kranz et al. [101]. 

## 3. Materials and Methods

### 3.1. Study Site

**Figure 10:**
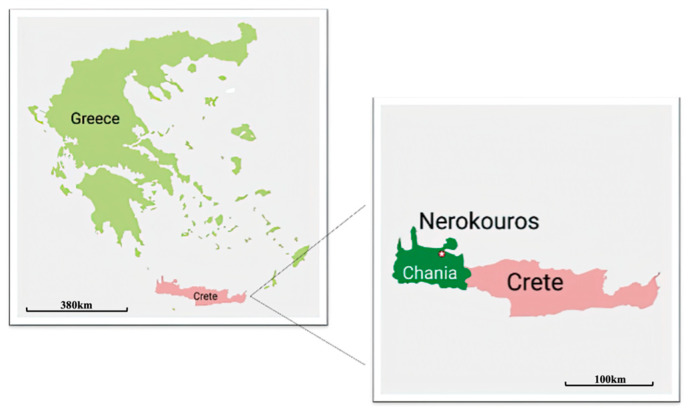
Experimental field of the olive grove at Nerokourou, western Crete, Greece. The star refers to the field’s accurate location.

**Figure 11:**
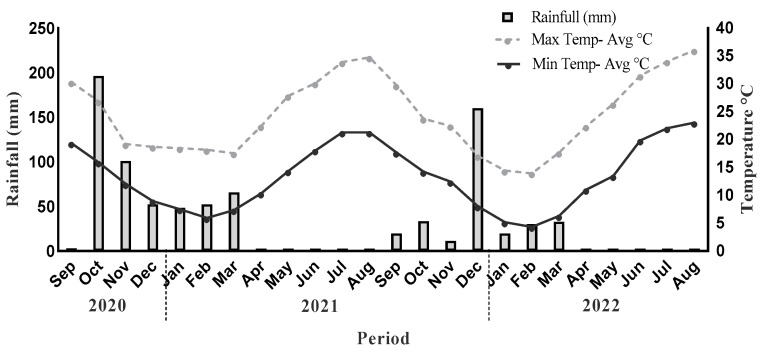
Climate trend of the experimental area during the two years of experimentation.

The research was carried out in a long-term experiment at the olive grove of the Institute of Olive Tree, Subtropical Crops and Viticulture. The experimental field is located in Nerokourou village, 51 m above sea level (35°28′36.76″ N-24°02′36.44″ E-51 m) (Figure 10) in the Prefecture of Chania, a typical olive-growing area in the south of Greece. The study site is situated in a primarily Mediterranean climate zone with a mean annual temperature of 18 °C. In general, the area experiences dry precipitation, with an annual average of 483 mm, and has a relative humidity of 64%. Key meteorological parameters, such as air temperature, rainfall, and humidity, were measured daily by a standard weather station placed close to the trial area during the two-year study (Figure 11).

### 3.2. Experimental Design

The experimental olive grove covers a total area of 1.1 hectares with a density of 7 × 7 m and belongs to the ‘Kalamon’ variety, which is a table olive cultivar. When it comes to management practices, this field has been following standard cultivation practices for around 45 years. Those olive-grove-management practices have ranged from intensive to no-tillage and have included a standard pruning protocol for canopy management, irrigation with drip systems, organic fertilizers, and mass trapping for olive-fly control. 

The soil analyses performed before the establishment of the trial revealed a sandy-loam soil (Clay 6.8%, Silt 28.0%, Sand 65.2%) with substantially low macro-elements contents at 0–40 cm depth (7.24 mg kg^−1^ NO_3_^−^-Ν, 8.53 mg kg^−1^ available P, and 72 mg kg^−1^ ex. K). The pH was found to be equal to 7.2. 

**Figure 12:**
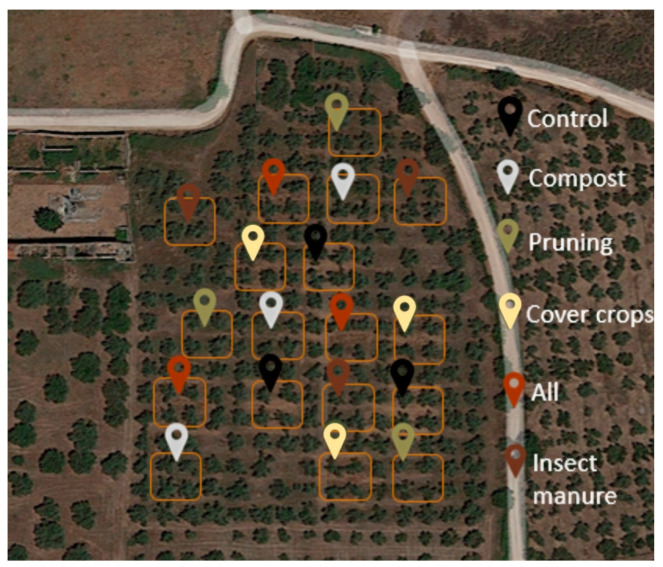
Experimental design adopted during the study period 2020–2021 and 2021–2022.

The study employed a randomized block design with three replications for each treatment, resulting in a total of 18 plots, each one having an approximate area of 200 m². The plots comprised four trees, surrounded by strip trees to avoid any interference between treatments (Figure 12). The field was homogeneous in terms of canopy size and natural vegetation, and the soil and landscape types were relatively uniform concerning their chemical and physical properties. Six treatments are defined in Table 1 and Table 2.

More detailed information concerning the amounts of plant biomass left on the field can be found in [102]. 

### 3.3. Soil Sampling and Analysis

To characterize the soil physicochemical properties of the experimental site, a representative set of soil samples was collected from each plot directly below the tree profiles, taking into account the different soil depths of 0–10 cm and 10–30 cm. After eliminating the bryophyte layer, two composite soil samples (at 0–10 cm and at 10–30 cm, each one formed by three subsamples) were taken from each plot at the designated depths by using a one-piece open-face auger. In total, 72 soil samples were collected during the field measurements, half of them on 25 May 2021 and the other part on the 9 May 2022. Sampling locations, each measuring 30 cm in diameter, were strategically positioned around the irrigation line drippers and within the tree rows. These locations were arranged in a pattern parallel to the irrigation line to guarantee a reliable number of samples and a detailed comprehensive assessment of soil physicochemical properties and water distribution. 

Before being analyzed, the composite soil samples of 1–1.5 kg were air-dried for three days at room temperature. Subsequently, the samples were disintegrated using a ceramic pestle and mortar, passed through a stainless-steel sieve with a mesh size of 2 mm, and then stored at ambient room temperature. For soil physicochemical analyses, only the 2 mm fraction of the soil was used. To assess the gravimetric soil moisture content, soil samples were collected at a depth of 0–30 cm. The measurements were taken at the end of July and September 2021 and also at the end of January and March 2022 to capture the soil moisture-content variability under different meteorological and management conditions.

The analysis of the chosen soil physicochemical properties in the study adhered to standard laboratory procedures. EC and pH were measured in a deionized water–soil solution with a calibrated pH meter and calibrated conductometer (Corwin and Rhoades 1982) [103], respectively. The determination of calcium carbonate (CaCO_3_) was based on qualitative analysis followed by quantitative analysis using a Bernard calcimeter. The Walkley and Black chromic acid wet oxidation method is used to determine soil carbon matter. The method involves adding to dried soil potassium dichromate (K_2_Cr_2_O_7_) 1 N and concentrated sulphuric acid (H_2_SO_4_ > 96%), then deionized water, sodium fluoride, phosphoric acid (H_3_PO_4_ > 85%), and a color indicator diphenylamine (DPA). The remaining dichromate is titrated with ferrous sulfate (FeSO_4_·7H_2_O) 0.5 N to determine the percentage of total carbon oxidized, which is used to calculate the percentage of SOM following this formula [104]:

Organic matter % = Organic C % × 1.754

To measure soil content in nitrate, soil samples were mixed with KCL (1 M) and analyzed using the cadmium reduction method (Keeney and Nelson 1982) [105]. The NO_3_⁻ concentration was then measured using a spectrophotometer at a wavelength of 500 nm after the addition of the nitrogen–nitrate reagent set, allowing time for color development. Available P was determined by sodium hydrogen carbonate extraction by mixing soil and active carbon with sodium bicarbonate 0.5 M (NaHCO_3_). A sample of the filtrate was combined with hydrochloric acid (HCL), phosphate, indophenol indicator, and sulfuric acid 5 N (H_2_SO_4_). The phosphorus concentration was measured using a Hitachi 1100 visible-UV spectrophotometer (Hitachi High-Tech Corporation, Ibaraki, Japan) at a wavelength of 882 nm after the addition of Reagent B (ascorbic acid/molybdate reagent), allowing the development of a blue coloration. The ammonium acetate method described by Doll and Lucas (1973) [106] was used to quantify the concentrations of exchangeable K, Ca, and Mg in soil extracts. Dried and ground soil samples were mixed with ammonium acetate buffer CH_3_COONH_4_ (1 M) and then analyzed using a pre-calibrated inductively coupled plasma optical emission spectrometer (ICP-OES) to determine the concentrations of Ca, and Mg, which were reported in units of parts per million (ppm).

The collected soil samples were freshly weighted (SF) and dried (SD) in the oven at 65 °C to a constant weight for 4 days, then weighted to calculate soil moisture (%), and each were calculated as follows:

Soil moisture content % = (SF − SD)/SD × 100

### 3.4. Statistical Analysis

Because of the complex interactions observed, the data analysis was realized separately for each year and each soil depth. Comparison between the six agricultural treatments (COMP, COVER, PRUN, INS, ALL, and CONT) was performed following a one-way analysis of variance (ANOVA) including as a factor soil-management treatment (six levels) for two periods (2020–2021 and 2021–2022), and for two soil depths (0–10 cm and 10–30 cm). The comparison was carried out at a level of significance of 0.05 using the SPSS 22 version for Windows. 

Then, the means of the treatments were separated by a least significant difference test (LSD) declared for all the elements and the Tukey test for soil water content at the (*p* < 0.05) significant level.

## 4. Conclusions

Employing agroecological practices that encompass the reutilization of agricultural waste, including techniques like soil mulching with composted olive-mill waste, insect manure, and chopped pruning residue, all without the need for mechanical soil disturbance, holds significant promise in enhancing soil fertility. This stands in contrast to an olive-grove-management system that involves no soil covering, solely relies on soil tilling, and lacks the incorporation of organic materials, which is less effective in promoting soil fertility.

Toward a transition to a circular bio-economy, the transition to different sustainable management approaches used in this study would have a positive long-term impact on climate change, rural stakeholders, and ecosystems. Our results indicate that combining agroecological practices as in the case of ALL has a promising impact on soil physiochemical properties for a short-term period in contrast to CONT or even PRUN, COVER, and COMP alone. 

Moreover, those practices bring significant amounts of mineral nutrients compared to natural weeds, and they will gradually release them into the soil during decomposition for a higher effect over a long-term period. 

Comparing sustainable and conventional managements of an olive grove offered more detailed information about the role of some agroecological practices and their synergistic action in improving soil quality and fertility. Similar studies should be conducted for long-term periods to allow for a better understanding of the changes affecting the whole agroecosystem. In addition, an optimization study of suitable quantities to apply could be included to encourage the vulgarization of these combined agroecological practices. 

## Figures and Tables

**Table 1:** Characteristics of the six treatments applied in the experiment with the application’s rate and date.

Treatment	Characteristics	Rate	Date
Compost (Comp)	From olive mill by-products (olive pomace, leaves, and liquid waste mixed with chopped pruning residue) No incorporation into the soil C/N ratio of 18:1; pH of 7.8; contained 49.76% total C, 2.77% total N, 2.26% total K, and 0.18% total P on a dry matter basis	12.5 t ha^−1^ 7.5 t ha^−1^ Both as dry matter	December 2020 November 2021
Cover crops (Cover)	A mixture of four annual pasture legumes *Pisum sativum* subsp. *Arvense*, *Medicago sativa* L., *Vicia faba* L., and *Vicia sativa* L. and *Avena sativa* L. as a grain. Seeded manually and incorporated into the soil using a tractor with a rotary tiller pass The vegetation was mechanically managed and left on the ground as mulch after most of the legume seeds reached physiological maturity	41.65 kg ha^−1^, 41.65 kg ha^−1^, 50 kg ha^−1^, 33.35 kg ha^−1^, and 25 kg ha^−1^, respectively	December 2020 December 2021
Pruning residues (Prun)	Chopped pruning residues (leaves and twigs up to 7 cm in diameter) from olive trees of the same grove Added to the soil as they are, as a mulch, without any tillage Contained between 51–55% total C, 0.6–1.8% total N, 0.4–1.2% total K, and 0.05–0.14% total P on a dry matter basis	11.55 t ha^−1^ per year (dry weight).	December 2020 December 2021
Insect manure (Ins)	From insect digestates (excreta of Tenebrio molitor fed with organic plants) Insect amendment to the soil without tillage C/N ratio of 10:1; pH of 5.8; contained 80% total C, 2.98% total N, 2.37% total K, and 1.84% total P on a dry matter basis	1.1 t ha^−1^ 1.2 t ha^−1^ Both as dry matter	December 2020 December 2021
Comp + Cover + Prun (All)	Involved the application of all the practices described above except the insect manure (compost, pruning residues, and cover crops) in the same plot	The corresponding rate of each treatment’s application rate	The corresponding periods of each treatment’s application date
Control (Cont)	Soil tillage with no organic matter additions Soil covered by natural vegetation Receiving only fallen olive-tree leaves and branches.	_	_

**Table 2:** Two years’ contribution of recycling materials in the budget of carbon (C), nitrogen (N), phosphorus (P), and potassium (K). Calculation was based on the amounts of the materials applied and their corresponding content in C, N, P, and K as cited in Table 1.

Treatment	Year	Contribution (kg ha^−1^ year^−^¹)				
		Dry Biomass	C	N	P	K
Compost (Comp)	2020 2021	12,500 7500	6220 3732	346.25 207.75	22.50 13.50	282.50 169.50
Cover crops (Cover)	2020 2021	8318 8436	2337 2303	207.95 227.77	24.95 24.46	228.70 240.43
Pruning residues (Prun)	2020 2021	11,550 11,550	6121 6121	124.74 124.74	92.40 92.40	11.27 11.27
Insect manure (Ins)	2020 2021	1100 1200	880 960	32.78 35.76	20.24 22.08	26.07 28.44
Comp + Cover + Prun (All)	2020 2021	32,318 27,436	14,678 12,156	678.94 560.26	139.85 130.36	522.47 421.20

## Data Availability

All data generated or analyzed during this study are included in this published article.
